# Broncho-alveolar inflammation in COVID-19 patients: a correlation with clinical outcome

**DOI:** 10.1186/s12890-020-01343-z

**Published:** 2020-11-16

**Authors:** Laura Pandolfi, Tommaso Fossali, Vanessa Frangipane, Sara Bozzini, Monica Morosini, Maura D’Amato, Sara Lettieri, Mario Urtis, Alessandro Di Toro, Laura Saracino, Elena Percivalle, Stefano Tomaselli, Lorenzo Cavagna, Emanuela Cova, Francesco Mojoli, Paola Bergomi, Davide Ottolina, Daniele Lilleri, Angelo Guido Corsico, Eloisa Arbustini, Riccardo Colombo, Federica Meloni

**Affiliations:** 1 Research Laboratory of Lung Diseases, Section of Cell Biology, IRCCS Policlinico San Matteo Foundation, 27100 Pavia, Italy; 2 Division of Anaesthesiology and Intensive Care, ASST Fatebenefratelli Sacco, Luigi Sacco Hospital, University of Milan, Milan, Italy; 3 University of Pavia and Pneumology Unit, IRCCS Policlinico S. Matteo Foundation, Pavia, Italy; 4 Center for Inherited Cardiovascular Diseases, IRCCS Policlinico San Matteo Foundation, University of Pavia, Pavia, Italy; 5 Pneumology Unit, IRCCS Policlinico S. Matteo Foundation, Pavia, Italy; 6 Molecular Virology Unit, Microbiology and Virology Department, IRCCS Policlinico S. Matteo Foundation, Pavia, Italy; 7 Division of Rheumatology, University of Pavia and IRCCS Policlinico S. Matteo Foundation, Pavia, Italy; 8 Department of Diagnostic Medicine, IRCCS Policlinico S. Matteo Foundation, Pavia, Italy; 9 Department of Clinical-Surgical, Diagnostic, and Pediatric Sciences, Unit of Anaesthesia and Intensive Care, University of Pavia, Pavia, Italy; 10 Laboratories of Genetics, Transplantology and Cardiovascular Diseases, and Biotechnology Laboratories, IRCCS Policlinico San Matteo Foundation, Pavia, Italy

**Keywords:** Bronchoalveolar lavage, COVID-19, Cytokines, Lung, SARS-CoV-2

## Abstract

**Background:**

Severe acute respiratory syndrome coronavirus 2 (SARS-CoV-2) rapidly reached pandemic proportions. Given that the main target of SARS-CoV-2 are lungs leading to severe pneumonia with hyperactivation of the inflammatory cascade, we conducted a prospective study to assess alveolar inflammatory status in patients with moderate to severe COVID-19.

**Methods:**

Diagnostic bronchoalveolar lavage (BAL) was performed in 33 adult patients with SARS-CoV-2 infection by real-time PCR on nasopharyngeal swab admitted to the Intensive care unit (ICU) (*n* = 28) and to the Intermediate Medicine Ward (IMW) (*n* = 5). We analyze the differential cell count, ultrastructure of cells and Interleukin (IL)6, 8 and 10 levels.

**Results:**

ICU patients showed a marked increase in neutrophils (1.24 × 10^5^ ml^− 1^, 0.85–2.07), lower lymphocyte (0.97 × 10^5^ ml^− 1^, 0.024–0.34) and macrophages fractions (0.43 × 10^5^ ml^− 1^, 0.34–1.62) compared to IMW patients (0.095 × 10^5^ ml^− 1^, 0.05–0.73; 0.47 × 10^5^ ml^− 1^, 0.28–1.01 and 2.14 × 10^5^ ml^− 1^, 1.17–3.01, respectively) (*p* < 0.01). Study of ICU patients BAL by electron transmission microscopy showed viral particles inside mononuclear cells confirmed by immunostaining with anti-viral capsid and spike antibodies. IL6 and IL8 were significantly higher in ICU patients than in IMW (IL6 *p* < 0.01, IL8 *p* < 0.0001), and also in patients who did not survive (IL6 *p* < 0.05, IL8 *p* = 0.05 vs. survivors). IL10 did not show a significant variation between groups. Dividing patients by treatment received, lower BAL concentrations of IL6 were found in patients treated with steroids as compared to those treated with tocilizumab (*p* < 0.1) or antivirals (*p* < 0.05).

**Conclusions:**

Alveolitis, associated with COVID-19, is mainly sustained by innate effectors which showed features of extensive activation. The burden of pro-inflammatory cytokines IL6 and IL8 in the broncho-alveolar environment is associated with clinical outcome.

**Supplementary Information:**

The online version contains supplementary material available at 10.1186/s12890-020-01343-z.

## Background

Severe acute respiratory syndrome coronavirus 2 (SARS-CoV-2), detected in Wuhan (China) in December 2019 [1], spread rapidly around the world, reaching pandemic proportions in March 2020. In Italy, from February up to June 17th, 237,500 SARS-CoV-2-positive adults have been documented with 34,405 deceased patients (http://www.salute.gov.it/portale/nuovocoronavirus/dettaglioNotizieNuovoCoronavirus). Since December 2019, researchers and clinicians have been trying to find out the pathogenic features of the new disease caused by SARS-CoV-2, named COVID-19, in order to develop an effective therapy. The typical clinical manifestation is severe pneumonia with hyperactivation of the inflammatory cascade and progression to acute respiratory distress syndrome (ARDS) in about 30% of patients [2–4]. As of yet, most research papers have focused on the inflammatory status at the plasma level of COVID-19 patients [3–7]. However, because the main target organ is the lung, it is crucial to understand the inflammatory status at the deep lung level during different stages of the infection. Currently, limited data are available about alveolar inflammatory status in COVID-19 patients because of concerns in relation to using bronchoscopy to avoid aerosol generation.

Herein, we aim to define innate inflammatory environment by the analysis of BAL cell differentials, ultrastructural cell analysis and cytokine profile in patients with severe COVID-19, comparing them with patients admitted to the intermediate medicine ward (IMW) enrolled by two major COVID-19 centers of Italy: Luigi Sacco Hospital (Milan, Italy) and the IRCCS Policlinico San Matteo Foundation Hospital (Pavia, Italy). Our hypothesis is that alveolar inflammation is mainly driven by innate immune effectors and correlate with clinical outcome of patients.

## Methods

### Patients

This study included 33 adults positive for SARS-CoV-2 infection, diagnosed by real-time PCR on nasopharyngeal swab. Twenty-eight patients with severe ARDS requiring intensive respiratory support (mechanical ventilation or extracorporeal support) were admitted to the Intensive Care Unit (ICU) at the Luigi Sacco Hospital (Milan, Italy) (*N* = 25), or the IRCCS Policlinico San Matteo Foundation Hospital (Pavia, Italy) (*N* = 3) (henceforth referred as ICU pts). The remaining 5 patients had moderate pneumonia with PaO_2_/FiO_2_ ratio > 250 and were admitted to the Intermediate Medicine ward (IMW) (*N* = 5) of the IRCCS Policlinico San Matteo Foundation. Failure of a trial with continuous positive airway pressure via a helmet was the indication for admission in ICU. Failure was defined as respiratory rate > 30 breaths per minute and PaO_2_ to FiO_2_ ratio < 150, or respiratory acidosis with pH < 7.36 and PaCO_2_ > 50 mmHg, or agitation, or confusion.

Research and data collection protocols were approved by the Institutional Review Boards (Comitato Etico di Area 1) (prot. 20,100,005,334) and by IRCCS Policlinico San Matteo Foundation Hospital (prot. 20,200,046,007). Written informed consent was obtained by all conscious patients. For unconscious patients the inform consent was obtained after recovery and for non-survivors was waived in accordance to the Italian law (Decreto legislativo 211/2003 art 5) (https://www.aifa.gov.it/documents/20142/1180150/2018-10-24_Ancona_Aita_24.10.18.pdf/f854902d-f5f6-f43d-65ba-746db9d92462).

### Clinical variables included in the analysis

Demographic and clinical characteristics of the enrolled patients were recorded in a dedicated database. Among the laboratory findings recorded during hospitalization, we took into account for the present study the following variables that are assessed at time of BAL sampling and reported in Table 1: PaO_2_/FiO_2_ ratio; white cell count (cells·ml^− 1^); lymphocyte count (cells·ml^− 1^); hemoglobin (cells·ml^− 1^); platelet count (cells·ml^− 1^); lactate dehydrogenase (LDH) (mU ml^− 1^); creatine kinase (CK) (mU ml^− 1^); D-dimer (ug l^− 1^); ferritin (ng ml^− 1^); albumin (g l^− 1^); c-reactive protein (CRP) (mg dl^− 1^); procalcitonin (ng ml^− 1^); and serum Interleukin-6 (IL6) (ng l^− 1^) when available. Ventilator settings and therapies such as corticosteroids, antiviral drugs, IL-6 antagonists or hydroxychloroquine or other antivirals were also recorded and reported in Table 2.

**Table 1:** Demographic characteristics of the studied population and laboratory findings at admission

	IMW (***N*** = 5)	ICU (***N*** = 28)	***P*** value
**Age (years)**^a^	64.0 (58.0–66.0)	59.5 (50.0–64.5)	0.2725
**Dead – N(%)**	0 (0)	12 (42.8)	0.1329
**Male gender –** N(%)	4 (80.0)	22 (78.6)	> 0.999
**Smoking history** – N(%)			
- Never smoked	0 (0)	24 (85.7)	0.0005^§^
- Former smoker	5 (100)	3 (10.7)	0.0002^§^
- Current smoker	0 (0)	1 (3.6)	> 0.999
**Comorbidities** – N(%)			
- Any	5 (100)	14 (50.0)	0.0574
- Arterial hypertension	2 (40.0)	6 (21.4)	0.5736
- Chronic heart disease	2 (40.0)	3 (10.7)	0.1546
- Cerebrovascular disease	0 (0)	1 (3.6)	> 0.999
- Chronic obstructive lung disease	0 (0)	1 (3.6)	> 0.999
- Lung transplant	3 (60.0)	0 (0)	0.0018^§^
- Diabetes mellitus type II	1 (20.0)	2 (7.1)	0.3996
- Cancer	1 (20.0)	1 (3.6)	0.2841
- Immunosuppression	4 (80.0)	2 (7.1)	0.0017^§^
**Symptoms –** N(%)			
- Fever	5 (100)	27 (96.4)	> 0.999
- Dyspnea, cough	1 (20.0)	28 (100)	0.0001^§^
- Diarrhea, nausea	2 (40.0)	2 (7.1)	0.0993
- Dysgeusia, anosmia	1 (20.0)	0(0)	0.1515
**Days between symptoms onset and hospitalization**^a^	7.0 (3.0–11.0)	6.0 (4.0–9.0)	0.9589
**Days in CPAP prior intubation**^a^	–	2.0 (1.0–6.5)	na
**Days between symptoms onset and BAL sampling**^a^	8.0 (4.0–12.0)	25.0 (20.0–30.0)	0.6852
**SOFA score**^ad^	–	12.0 (8.0–13.0)	na
**Laboratory findings**^ad^			
- PaO_2_/FiO_2_ ratio	333.4 (195.5–379.1)	121.0 (97.5–157.5)	0.0060^§^
- White cell count (cells·ml^− 1^)	7720.0 (4920.0–11,890.0)	13,700.0 (9400.0–16,810.0)	0.0379^§^
- Lymphocyte count (cells·ml^− 1^)	900.0 (470.0–1990.0)	1130.0 (760.0–1675.0)	0.5807
- Hemoglobin (cells·ml^−1^)	10.7 (9.8–12.8)	10.05 (9.2–10.8)	0.2997
- Platelet count (cells·ml^− 1^)	278.0 (158.0–368.0)	353.0 (176.0–361.0)	0.9586
- LDH (mU ml^− 1^)	269.0 (171.5–339.0)	414.5 (419.5–547.8)	0.0169^§^
- CK (mU ml^− 1^)	120.0 (46.0–582.5)	66.5 (32.5–419.8)	0.5368
- D-dimer (ug l^−1^)	1077.0 (689.0–1465.0)	3299.0 (1599.0–5497.0)	> 0.999
- Ferritin (ng ml^− 1^)	1191.5 (475.3–1928.0)	1413.0 (298.8–2534.0)	0.8081
- Albumin (g l^− 1^)	3.1 (2.4–3.6)	2.0 (2.0–2.1)	0.0015^§^
- CRP (mg dl^− 1^)	11.3 (3.2–12.1)	36.1 (15.4–137.6)	0.0228^§^
- Procalcitonin (ng ml^− 1^)	0.1 (0.1–1.1)	0.4 (0.3–1.0)	0.2135
- Serum IL6 (ng l^−1^)^b^	41.1 (13.4–72.5)	476.5 (110.6–1636.0)	0.0102^§^
**Bacterial co-infections -** N(%)^c^	2 (40.0)	21 (75.0)	0.2911
**Fungal co-infections -** N(%)^c^	0 (0)	4 (14.3)	0.5997
**Viral co-infections -** N(%)^c^	0 (0)	1 (3.6)	> 0.999

§significant difference (*p* < 0.05 with respect to IMW)

^a^Data are expressed as median and interquartile range (IQR)

^b^Data regarding serum IL6 levels are missing for 12 ICU patients (42.8%)

^c^All pathogens were isolated on BAL

^d^ Referred to the same day of BAL

**Table 2:** Treatment characteristics in ICU patients

Treatment	Survivors (***N*** = 16)	Non-survivors (***N*** = 12)	***P*** value
**Tocilizumab N(%)**	4 (25)	4 (25)	0.6908
**Remdesivir N(%)**	3 (18.8)	4 (5)	0.6618
**Hydroxychloroquine N(%)**	12 (75)	12 (100)	0.1131
**Ritonavir/Lopinavir N(%)**^**a**^	7 (50)	9 (75)	0.1358
**Corticosteroids N(%)**^**b**^			
- Dexamethasone 0.4 mg·Kg^− 1^	4 (25)	5(42)	0.4319
- Methylprednisolone 0.3 mg·Kg^−1^	0 (0)	1(8.3)	0.4286
- Hydrocortisone 150 mg·die^−1^	1 (6.25)	1(8.3)	> 0.999
**Mechanical ventilation**^**c**^			
- Vt/IBW -ml Kg^−1d^	7.85(7.6–8.85)	6.4 (6.1–8)	0.4275
- PEEP -cmH_2_0	11(8–13.25)	12 (8–12)	0.9073

^a^Data regarding the administration of Ritonavir/Lopinavir are missing for two patients who died and two who survived (14.8%)

^b^Data regarding the administration of steroids is missing for one dead patient (3.70%)

^c^Data regarding ventilation parameters are missing of two dead patients (7.41%)

^d^*Vt/IBW* Tidal volume/ideal body weight calculated using the gender-specific Acute Respiratory Distress Syndrome Network (ARDSnet) formulas [8]

### BAL collection

All bronchoscopies were performed at both centers for diagnostic purposes in order to investigate the presence of other co-infections and no complications related to bronchoscopy occurred in patients [9].

BAL was collected from mechanically ventilated ICU patients according to the clinical requirement through disposable bronchoscope aScope™ 4 (Ambu A/S, Baltorpbakken, Denmark) according to WHO guidelines. Security procedures were strictly observed, and only essential personnel were involved in endoscopic exams. The Personal Protective Equipment included water-resistant gowns, gloves, respiratory protection (FFP3 mask), and eye protection. Six patients underwent repeated bronchoscopies. Therefore, the number of overall BAL samples were 45. When disposable bronchoscopes were not available, we followed the standard High-Level Disinfection for the utilization of re-usable bronchoscopes. BAL specimens were managed in a biosafety level 3 laboratory until inactivation. BALs were centrifuged at 400 g for 10 min at room temperature, inactivated with a 0.2% SDS and 0.1% Tween-20 solution followed by 65 °C for 15 min. BAL supernatants were then stored at − 20 °C until analysis. Cell pellets were fixed, then stained with Papanicolaou to analyze differential cell count or used for ultrastructural analysis.

### Ultrastructural analysis and immunostaining

BAL samples were fixed with Karnovsky’s fixative, treated with 1.5% OsO_4_ in 0.2 mol l^− 1^ cacodylate buffer (pH 7.3), dehydrated and embedded in Epon-Araldite resin. Ultrathin sections were stained with lead citrate and uranyl acetate and examined with a electron microscope (JEOL JEM-1011). SARS-CoV-2 infected VERO E6 cells at 48 and 72 h were used as positive controls.

The immunostaining of paraffin-embedded sections was done using SARS-CoV-2 (2019-nCoV) Nucleoprotein / NP Antibody, Rabbit MAb (Sino Biological, Catalog number: 40143-R019) – Dilution 1:1000 and SARS-CoV-2 (2019-nCoV) Spike Antibody, Rabbit MAb (Sino Biological, Catalog number: 40150-R007) - Dilution 1:400. The images were acquired with Nikon Eclipse Ti2 (software NIS-Elements AR 5.02.03).

### ELISA assays

To quantify IL8, IL10 and IL6, we used the SimpleStep ELISA® kit (Abcam, Cambridge, UK). Briefly, 50 μl of each sample was added to ELISA kit wells with the addition of 50 μl antibody cocktail. After 1 h at room temperature on a plate shaker, plates were washed three times to eliminate the unbound antibody. Substrate (100 μl) was incubated for 10 min in the dark at room temperature on a plate shaker, followed by 100 μl stop solution to read the absorbance at 450 nm. For IL8 and IL6, samples were diluted (from 1:10 to 1:1000). To assess that the inactivation protocol that we adopted did not alter the quantification of citokines, we firstly tested non COVID-19 infected BAL fluids with or without inactivation solution, obtaining the same quantification of all three citokines (data not shown).

### Statistical analysis

Comparison of cell counts between ICU and IMW was analyzed by multiple t-test followed by the Sidak method. Analysis of cytokines between ICU vs. IMW or survivors and non-survivors were carried out using the Mann-Whitney test. With regard to the analysis of cytokines according to treatment strategies we used the Kruskal-Wallis test followed by Dunn’s multiple comparison test. Association between variables was assessed with Spearman’s correlation. Statistical significance was defined as *p* ≤ 0.05. All data are represented as median (interquartile range – IQR). Data were statistically analyzed with Graphpad Prism version 8.4.1.

## Results

From March 7 to April 30, a total of 45 BAL samples were collected from 5 IMW and 28 ICU patients with proven COVID-19 infection. Patients characteristics are shown in Table 1, and data concerning administered treatment in Table 2. All patients from the IMW ward survived. Significant differences regarding laboratory abnormalities among ICU and IMW patients, when present, are shown in Table 1.

**Fig. 1:**
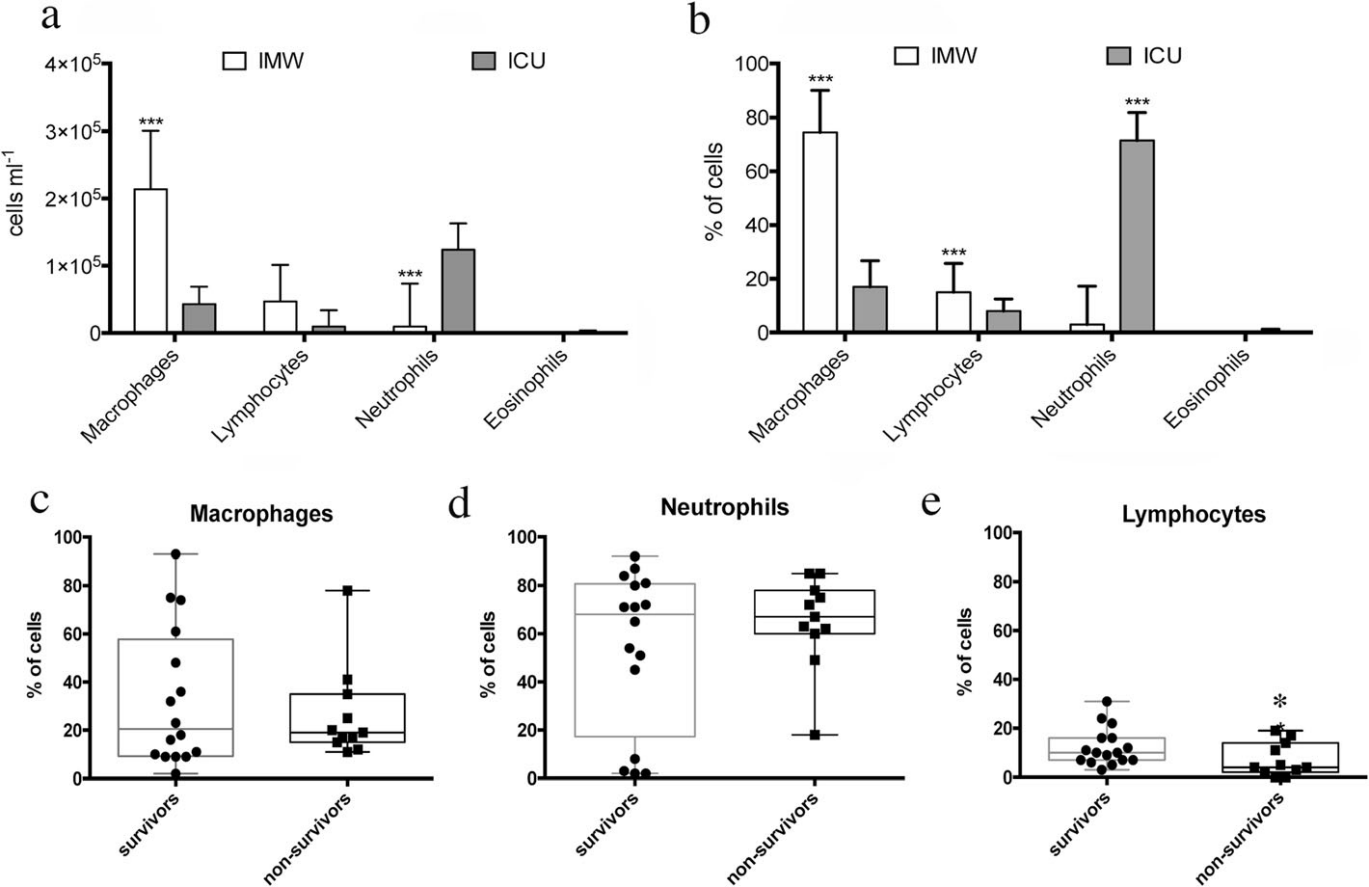
Cytological analysis of BAL collected. **a** Histograms of total cells ml^− 1^ counted in IMW and ICU samples showed as median (IQR). ***, *p* < 0.001 vs. ICU **b** Cell counts are converted in percentage of leucocytes comparing IMW with ICU samples. Data are shown as median (IQR). ***, *p* < 0.001 vs. ICU. **b**-**d** Percentage of each leucocyte quantified in all analyzed BALs dividing them into survivors and non-survivors. Data are shown as median (IQR). *, *p* < 0.05

Firstly, we assessed BAL cell differentials in ICU and in IMW patients that presented a median of total cells of 0.25 × 10^6^ ml^− 1^ (0.15–0.52) and 0.3 × 10^6^ ml^− 1^ (0.15–0.52), respectively (*p* > 0.999). A different leukocyte profile between ICU and IMW patients was observed (Fig. 1a and b). In particular, ICU patients showed a marked prevalence of neutrophils (1.24 × 10^5^ ml^− 1^, 0.85–2.07) with a lesser extent of macrophages (0.43 × 10^5^ ml^− 1^, 0.34–1.62) and lymphocytes (0.97 × 10^5^ ml^− 1^, 0.024–0.34) as compared to IMW patients, who had median macrophages at 2.14 × 10^5^ ml^− 1^ (1.17–3.01), lymphocytes at 0.47 × 10^5^ ml^− 1^ (0.28–1.01) and neutrophils at 0.095 × 10^5^ ml^− 1^ (0.05–0.73).

IMW patients presented a significant decrease of macrophages (*p* = 0.029) and a slight, but not significant, increase of lymphocyte (*p* = 0.09) and neutrophils (*p* = 0.11) with respect to normal reference values from our lab (data not shown) [10].

Dividing all patient samples into survivors and non-survivors, only for lymphocytes we observed a significant difference (*p* = 0.03) between two groups, where non-survivors showed less percentage of lymphocytes compared to survivors (Fig. 1e). Regarding macrophages and neutrophils (Fig. 1c and d), even if there was a trend towards a lower macrophage fraction among non-survivors, they did not reach a significant difference between two groups of analysis.

**Fig. 2:**
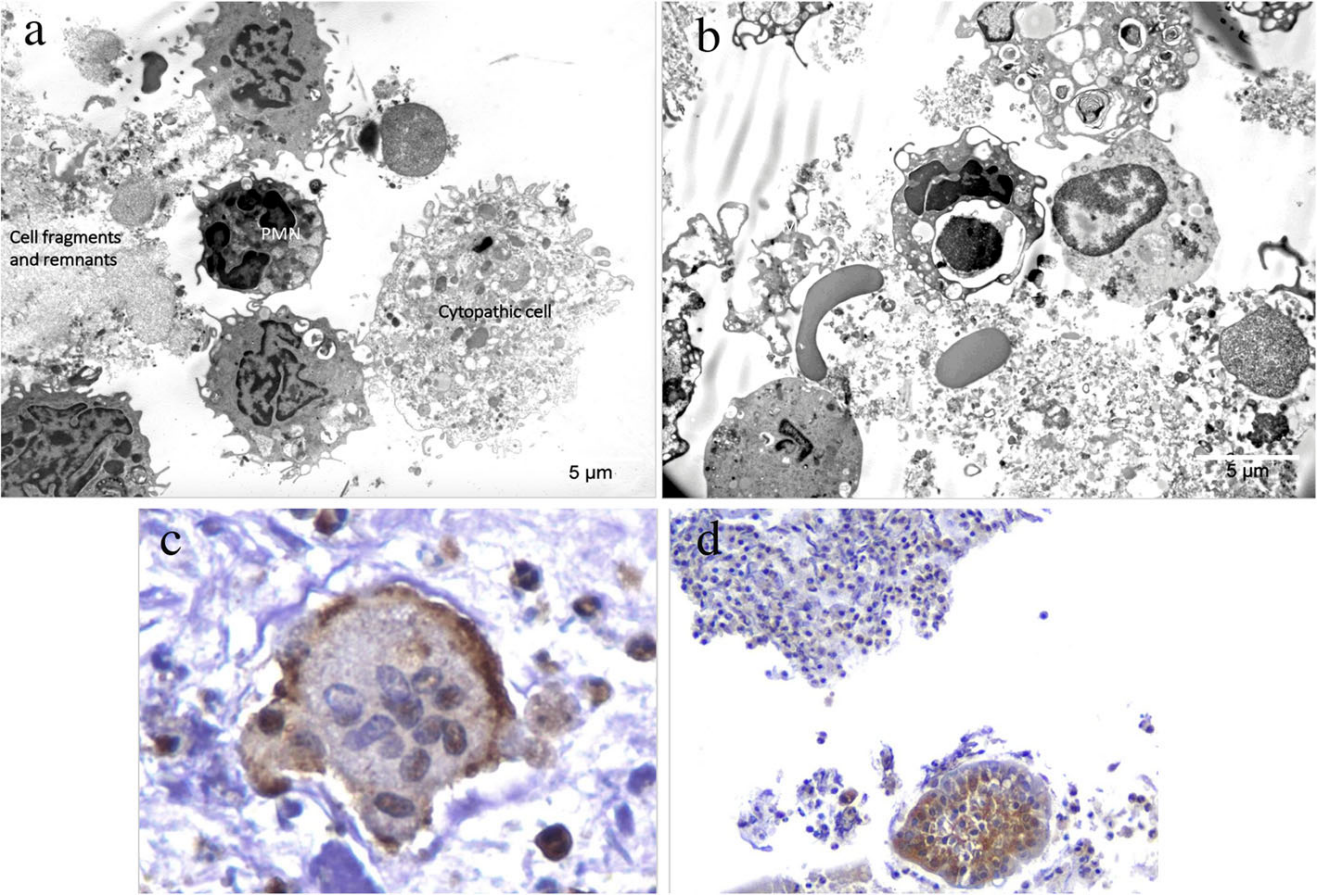
**a** and **b** Electron micrographs showing both **a** cytopathic cells and still morphologically preserved mononuclear cells and **b** extensive cytopathy involving all cells. **c** the SARS-CoV-2–anti-spike antibodies immunoreactive multinucleated cells in the BAL sample of one of the ICU patients, 40x; **d** flaps of epithelial cells and inflammatory cells immunoreacting with anti-spike antibodies, 10x. PMN = polymorphonuclear leukocyte. Scale bar = 5 μm

Ultrastructural study of ICU BAL samples showed many cytopathic cells, with loss of integrity of cell membranes and cytoplasm vacuolization (Fig. S1a-d). Mononuclear cells and neutrophils were detected with morphological features of activation (Fig. 2a and b). Viral particles, both single and in small clusters, were identified in numerous cells, particularly in cytopathic epithelial cells and mononuclear cells, with morphology (spikes) and size (80–120 nm) consisting with coronavirus (Figs. S1a-d and S2), confirmed by immunohistochemistry (Fig. S1e, f and Fig. 1c, d). Infected VERO E6 cells used as positive control showed large amounts of clustered and single viral particles clustered (Fig. S3).

**Fig. 3:**
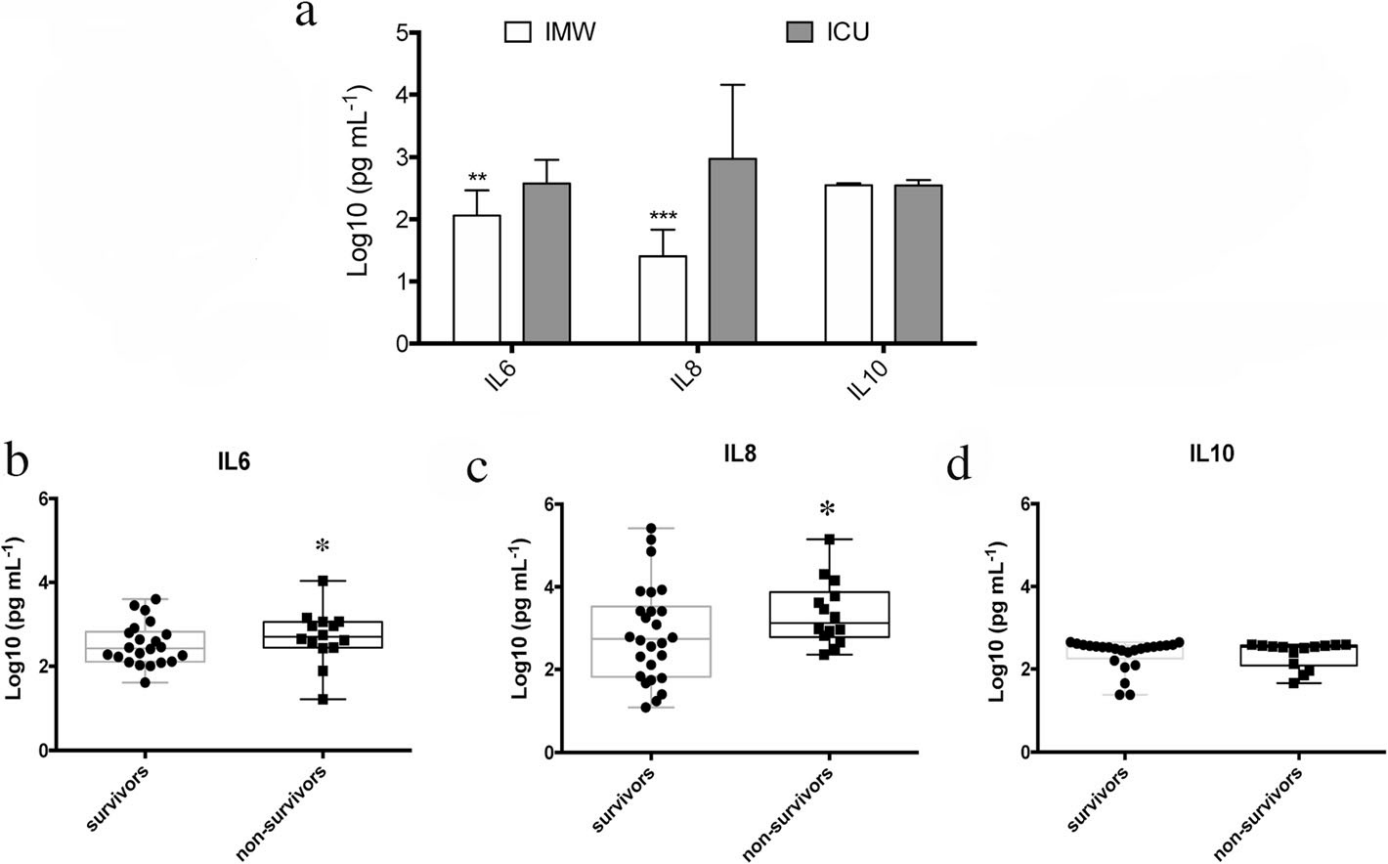
**a** Cytokines quantified in BAL of ICU patients and IMW patients (repeated BAL samples in the same patients have been excluded) has been transformed in Log10(pg ml^− 1^) and represented as median (IQR). **p < 0.01 vs. ICU; ***p < 0.001 vs. ICU. **b**-**d** Quantified cytokines divided in survivors and non-survivors. *p ≤ 0.05 vs. survivors. Data has been transformed as Log10(pg ml^− 1^) and represented as median (IQR)

The following cytokines: IL6, IL8 and IL10 were quantified on cell-free BAL by ELISA. Figure 3a shows the different distribution of three cytokine levels between ICU and IMW patients. Pro-inflammatory cytokines, IL6 and IL8, were significantly higher in ICU than in IMW patients (IL6 *p* = 0.006 and IL8 *p* < 0.0001), while IL10 did not show a significant difference between groups (*p* = 0.249). When analyzing cytokines according to outcome, higher IL6 (Fig. 3b, *p* = 0.027) and IL8 (Fig. 3c, *p* = 0.05) BAL levels were detectable in non-survivors with respect to survivors, with no difference for IL10 (Fig. 3d).

**Fig. 4:**
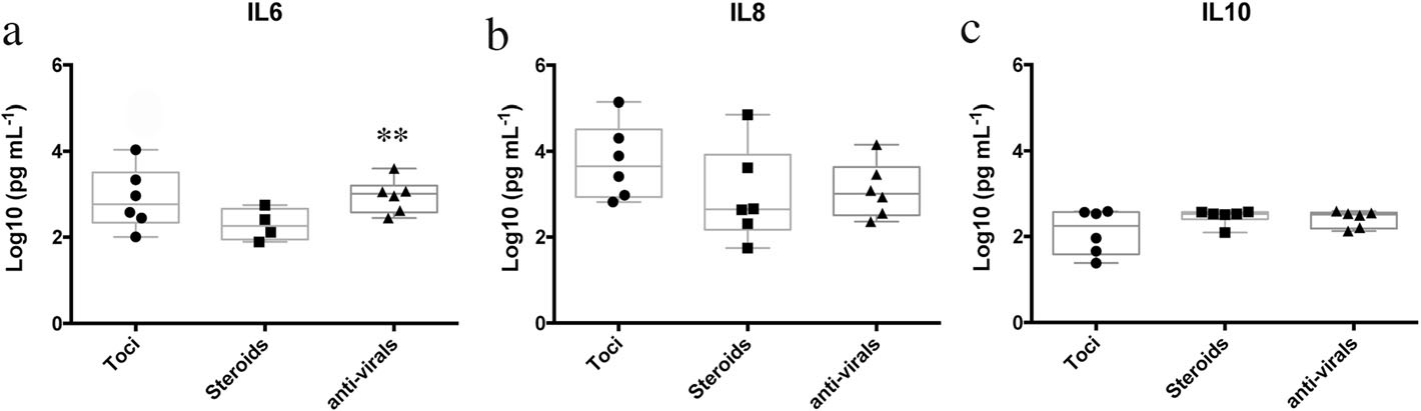
Differences between cytokines after tocilizumab, steroids, and anti-viral drugs treatment. Hydroxychloroquine has been added in the anti-viral group. Data are represented as median (IQR). **p < 0.05 vs. steroids

Variations of cytokine BAL levels according to treatment strategies were also evaluated. We decided to consider only BAL samples derived from patients who started specific treatment within 7 days prior to BAL sampling in order to assess the potential influence of treatment on cytokine levels. Moreover, considered patients were not submitted to other treatment regiments before BAL sampling. Due to the limited sample size, we restricted the analysis to tocilizumab, steroids and antiviral treatments (remdesivir, lopinavir/ritonavir) also including in this last group, hydroxychloroquine. Figure 4 shows that we observed a significant difference between different groups only in the case of IL6, in particular in patients treated with steroids that showed lower BAL IL6 compared to those who received or antiviral drugs (*p* = 0.030) (Fig. 4a).

**Fig. 5:**
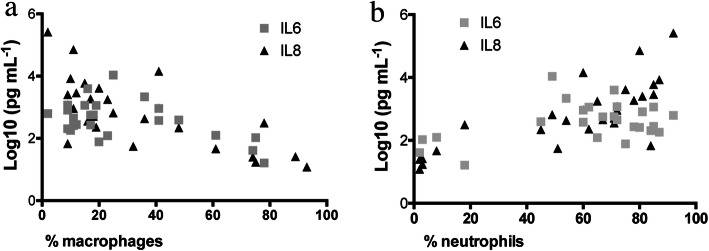
Correlation cytokine/BAL cell differentials: **a** macrophages percentage vs. IL6/IL8 and **b** neutrophil percentage vs. IL6/IL8

Finally, we investigated whether BAL cytokines correlated with differential cell counts quantified previously (Table S1) or between each other (Table S2), considering all collected BAL samples. We found that IL6 and IL8 correlated directly with neutrophils (Table S1 and Fig. 5b) and inversely with macrophages percentage (Table S1 and Fig. 5a). We observed that the level of IL8 also correlated inversely with lymphocytes percentage (Table S1). Analyzing the correlation between cytokines, levels of IL6 and IL8 were correlated between each other, while IL10 correlated inversely with IL8 (Table S1). These correlations between cytokines are clear in two representative cases of patients subjected to repeated bronchoscopies (Fig. S4).

To have a preliminary observation about pulmonary inflammation and its relationship to serum inflammation status, we analyzed correlation between CRP/IL6 BAL and IL6 serum/IL6 BAL. Regarding CRP/IL6 BAL we did not observe any direct correlation (spearman coefficient = − 0.038; *p* = 0.83). In contrast, for patients from whom we reported IL6 serum and BAL quantification sampling on the same day (20 from ICU and 4 from IMW), we analyzed the correlation and observed a direct correlation (Spearman coefficient = 0.53 with *p* < 0.01) (Fig. S5).

## Discussion

Up to now, there has been little information about the alveolar lung inflammatory status in SARS-CoV-2 patients, since most of the published studies assessed the inflammatory status of COVID-19 patients at peripheral level (plasma or serum) level [2–7]. In the present study we analyzed the broncho-alveolar inflammatory environment of patients admitted to the ICU and IMW of two hospitals situated in the epicenter of the Italian epidemic outbreak. In particular, we evaluated cell differentials, cell activation by morphological features, and cytokines profile on retrieved BAL fluids. Among cytokines, we decided to focus our attention on IL6, a well-known inflammatory cytokine that has been used as a marker of macrophages activation in the peripheral blood of COVID-19 affected patients [11]; IL8, a pro-inflammatory CXC chemokine that exerts specific chemotactic and activating functions on neutrophils; and IL10, an anti-inflammatory and immunoregulatory cytokine, produced by several immune effectors, whose role in SARS-CoV-2 infected patients is still in debate [12].

Thanks to our study, we observed that in severe ICU COVID-19 patients the alveolitis was associated to an hyperactivation of innate effectors, macrophages and neutrophils, that showed pseudopodia and cell-to-cell contacts (Fig. 2a and b). Moreover, a large number of cytopathic cells with vacuolization, osmiophilic bodies, loss of integrity of both nuclear and plasma membranes and cell fragmentation were present (Figs. S1 and S2 and Fig. 2). Cell differential analysis confirmed what was expected after peripheral blood analysis. In ICU patients there is an excessive infiltration of neutrophils at alveolar level, in contrast to IMW patients (Fig. 1a and b). This result is confirmed by autopsy studies that revealed high neutrophilic infiltration inside the alveolar space [13]. However, even if there is a significant difference between IMW and ICU of neutrophil fraction (Fig. 1a and b), we did not observe any correlation between neutrophils and clinical outcome (Fig. 1d).

Regarding lymphocytes, ICU patients revealed a strongly and significantly reduced lymphocytes percentage compared to IMW (Fig. 1), corroborating findings recently reported by Liao and colleagues who showed a decreased lymphocyte counts in severe patients with respect to the mild ones [14].

Concerning the three analyzed cytokines, we demonstrated that ICU patients are characterized by significant high levels of the two pro-inflammatory cytokines, IL6 and IL8, with respect to IMW patients (Fig. 3a). This result is sustained by previous reports obtained for Middle East Respiratory Syndrome (MERS) pneumonia patients, demonstrating in respiratory specimens high expression levels of inflammatory cytokines IL-1α and IL-1β and IL-8 (CXCL8) [15]. We not only demonstrated that the two pro-inflammatory cytokines levels are related to the severity of illness, but also with the outcome of patients. In fact, we demonstrated that upon analyzing survivors in comparison to patients who did not survived, significant lower level of IL6 (Fig. 3b) and IL8 (Fig. 3c) were observed in the survivor group with respect to non-survivors.

In order to understand if the treatment regimen could have an impact on inflammatory status at alveolar level, we evaluated cytokines in a small group of ICU patients who received one type of treatment only. The significant result was obtained only for BAL-IL6 when we compared patients submitted to a steroid course against patients who received anti-viral drugs (Fig. 4a). The same trend towards a decrease was also observed for IL8, without reaching a statistical significance (Fig. 4b).

Thus, taking together all our findings, we can suggest that steroids seem to be able to limit the secretion of IL6 in severe COVID-19. This anti-inflammatory effect could be correlated to a better outcome, in fact the clinical trial called “RECOVERY”, recently reported that dexamethasone reduced deaths by one-third in ventilated patients [16].

Regarding the role of IL10 in COVID-19 infection is a matter of ongoing debate. Increased serum levels of IL10 have been described in severe SARS-CoV-2 patients with respect to those affected by mild disease. Moreover, it has been demonstrated a direct correlation between IL10 and IL6 at serum levels, suggesting that both cytokines could be used as predictors of higher risk of disease deterioration [17, 18]. However, in another paper, McElvaney et al. 2020 demonstrated that IL10 response in plasma of SARS-CoV-2 patients is inadequate to the high levels of pro-inflammatory cytokines (IL6 and IL1-beta) [19]. Unfortunately, our present study does not contribute in clarifying this issue. In fact, even if we detected a high median BAL levels for IL10 (334 pg mL^− 1^, 133.2–372.1) in all patients, this cytokine was not associated to disease severity or to survival (Fig. 3). In addition, no significant correlation was found between IL10 and IL6 in BAL, while only a negative and significant correlation with IL8 levels was present (Table S2). Thus, on the basis of the present study, a role of BAL IL10 as a specific disease marker cannot be clearly inferred.

Lastly, we tried to understand if pulmonary inflammation correlates with peripheral one, but observing only a positive correlation between BAL and serum IL6, while BAL IL6 and serum CRP did not significantly correlate.

We believe that a significant role in driving lung inflammation in COVID-19 patients has to be ascribed also to IL8, that showed higher BAL values among ICU compared to IMW patients (Fig. 3a) and was associated to a negative outcome (Fig. 3c). IL8 acts as a specific chemoattractant and activating factor of neutrophils, in fact a direct correlation between BAL IL8 and neutrophils count is detectable in our patients (Table S1 and Fig. 5b). It has been already demonstrated that excessive neutrophil activation in the lung is associated to NETs formation and release of oxygen radicals and proteolytic enzymes, which can drive severe epithelial and endothelial injury [20]. Thus, modulation of IL8 pathway might represent a possible target of future intervention.

We are aware that this study has some limitations. We performed these preliminary analyses on limited sample size given the high risk of infection of the health care personnel. This limited our possibility to make further analyses, such as cell surface activation markers assessment by flow cytometry on macrophage or lymphocytes. Another limitation is the lack of paired assessment of cytokines in the peripheral blood due to the absence of stored serum samples from acute COVID19 patients in our institutions. Finally, we know that the infectious complications registered (Table 1) might have influenced cell differentials and cytokine levels however, the percentage of bacterial and fungal co-infection was very high in all patients in ICU without a significant difference between survivors and non-survivors.

## Conclusion

In conclusion, in present study, we contributed to define the broncho-alveolar inflammatory environment in acute COVID-19 patients represented by high innate effectors infiltration, in particular neutrophils, that correlate with two pro-inflammatory cytokines, IL6 and IL8, whose BAL level reflect the outcome of COVID-19 patients.

## Supplementary Information

**Additional file 1:** **Figure S1.** Severely cytopathic cells in BAL sample of ICU patient. (a) Squared area is enlarged in (b) that shows isolated viral particles in the cytoplasms; (c) shows a similar severe cytopathic cell: the squared area is enlarged in (d) with isolated viral particles. (e) BAL cells immunoreacting with anti-spike antibodies were variably represented in BAL samples (10x), including (f) ciliated epithelia, 20x. Scale bar = 2 μm and 200 nm. **Figure S2.** (a-b) Electron micrograph showing a ciliated cytopathic cell with single viral particles (enlarged in b) and vesicles containing smaller viral particles (upper white arrow in b). Scale bar = 1 μm and 200 nm. **Figure S3.** The figure shows infected VERO E6 cells at (a) 48 and (b-f) 72 h from infection with SARS-CoV-2. (a) severely cytopathic cell (lower) in close contact with a non-cytopathic cell (upper). Scale bar = 2 μm. (b) Extensively damaged cell with multiple viral arrays. Scale bar = 1 μm. (c) Viral particles with variable morphology, with and without spikes in cytoplasmic vesicles (➔) and free in the cytoplasm (squared area). Scale bar = 200 nm. (d) the extensive immunostaining with anti-spike antibodies in infected VERO E6 cells, 20x. (e) Spiked viral particles in cytoplasmic vesicles. Scale bar = 200 nm. (f) Spiked viral particles free in the cytoplasm. Scale bar = 100 nm. **Table S1.** Correlation analysis of entire BAL collected between cell populations and cytokines. **Table S2.** Correlation analysis between cytokines quantified in BALs overall. **Figure S4.** IL6, 8 and 10 trends in two representative ICU patients. (a) survivor (treated with anti-viral + corticosteroids) and (b) non-survivor (treated only with tocilizumab). **Figure S5.** Correlation between IL6 quantified in plasma vs. IL6 quantified in BAL of the same patients sampled at the same day. *r* = Sparman coefficient; *p* = *p* value.

## Data Availability

The datasets supporting the conclusions of this article are included within the article (and its additional file).

## Abbreviations

SARS-CoV-2: Severe acute respiratory syndrome coronavirus 2
ARDS: Acute respiratory distress syndrome
BAL: Bronchoalveolar lavage
IMW: Intermediate medicine ward
ICU: Intensive care unit
IL: Interleukin
CRP: C-reactive protein
LDH: Lactate dehydrogenase
CK: Creatine kinase
IQR: Interquartile range
