# Supplementary material for: Broncho-alveolar inflammation in COVID-19 patients: a correlation with clinical outcome
Source: BMC Pulm Med. 2020 Nov 16;20:301. doi: 10.1186/s12890-020-01343-z (PMC7668012; doi:10.1186/s12890-020-01343-z)
Supplement: Supplementary file 1 — Additional file 1: Figure S1. Severely cytopathic cells in BAL sample of ICU patient. (a) Squared area is enlarged in (b) that shows isolated viral particles in the cytoplasms; (c) shows a similar severe cytopathic cell: the squared area is enlarged in (d) with isolated viral particles. (e) BAL cells immunoreacting with anti-spike antibodies were variably represented in BAL samples (10x), including (f) ciliated epithelia, 20x. Scale bar = 2 μm and 200 nm. Figure S2. (a-b) Electron micrograph showing a ciliated cytopathic cell with single viral particles (enlarged in b) and vesicles containing smaller viral particles (upper white arrow in b). Scale bar = 1 μm and 200 nm. Figure S3. The figure shows infected VERO E6 cells at (a) 48 and (b-f) 72 h from infection with SARS-CoV-2. (a) severely cytopathic cell (lower) in close contact with a non-cytopathic cell (upper). Scale bar = 2 μm. (b) Extensively damaged cell with multiple viral arrays. Scale bar = 1 μm. (c) Viral particles with variable morphology, with and without spikes in cytoplasmic vesicles (➔) and free in the cytoplasm (squared area). Scale bar = 200 nm. (d) the extensive immunostaining with anti-spike antibodies in infected VERO E6 cells, 20x. (e) Spiked viral particles in cytoplasmic vesicles. Scale bar = 200 nm. (f) Spiked viral particles free in the cytoplasm. Scale bar = 100 nm. Table S1. Correlation analysis of entire BAL collected between cell populations and cytokines. Table S2. Correlation analysis between cytokines quantified in BALs overall. Figure S4. IL6, 8 and 10 trends in two representative ICU patients. (a) survivor (treated with anti-viral + corticosteroids) and (b) non-survivor (treated only with tocilizumab). Figure S5. Correlation between IL6 quantified in plasma vs. IL6 quantified in BAL of the same patients sampled at the same day. r = Sparman coefficient; p = p value. [file 12890_2020_1343_MOESM1_ESM.docx]

Supplementary material of:

**Broncho-alveolar inflammation in COVID-19 patients: a correlation with clinical outcome**

Pandolfi Laura^1^*°, Fossali Tommaso^2^*, Frangipane Vanessa^1^, Bozzini Sara^1^, Morosini Monica^1^, D’Amato Maura^1^, Lettieri Sara^3^, Urtis Mario^4^, Di Toro Alessandro^4^, Saracino Laura^5^, Percivalle Elena^6^, Tomaselli Stefano^5^, Cavagna Lorenzo^7^, Cova Emanuela^8^, Mojoli Francesco^9^, Bergomi Paola^2^, Ottolina Davide^2^, Lilleri Daniele^10^, Corsico Angelo Guido^3^, Arbustini Eloisa^4^, Colombo Riccardo^2^ and Meloni Federica^3^

^
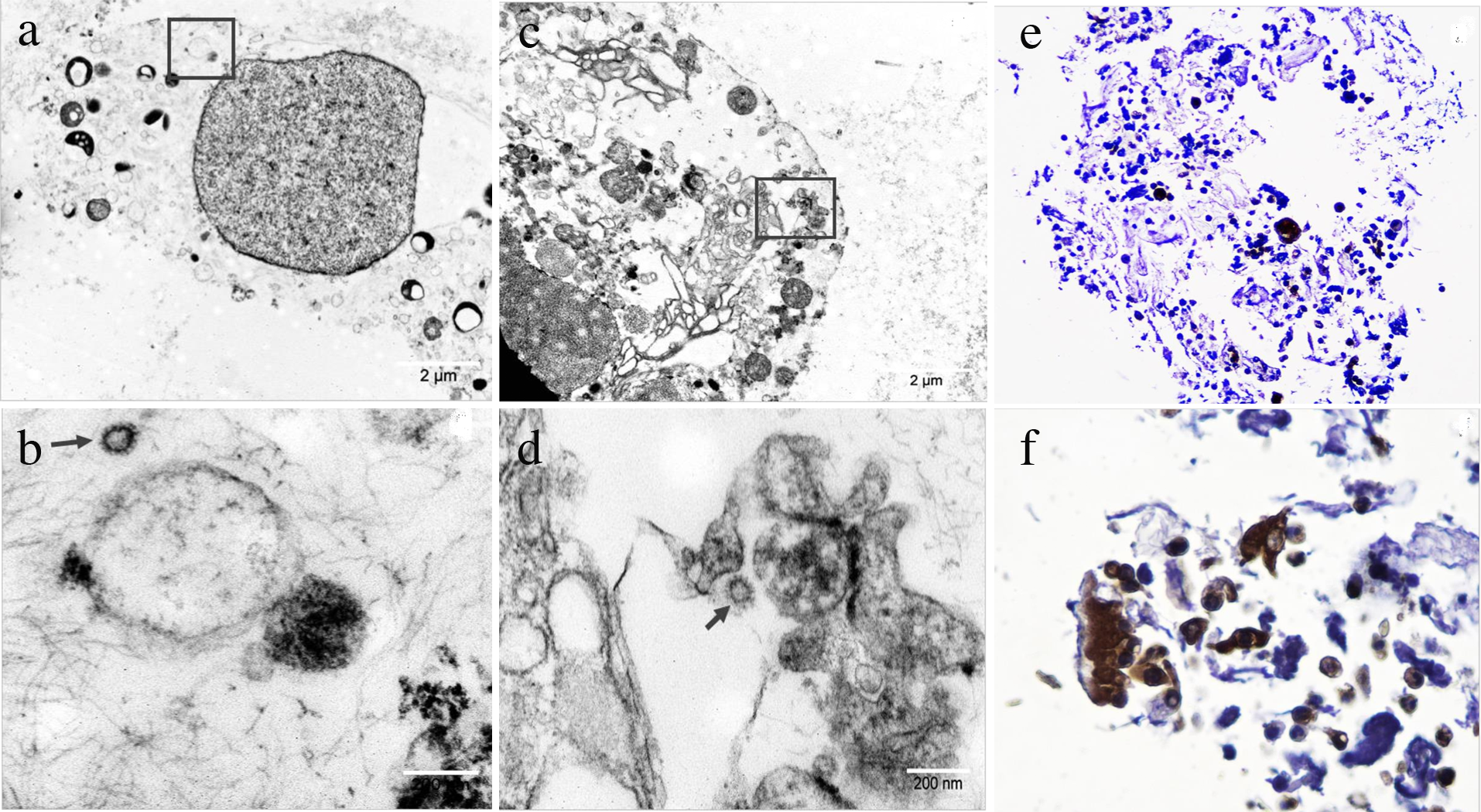
^

Figure S1. Severely cytopathic cells in BAL sample of ICU patient. (a) Squared area is enlarged in (b) that shows isolated viral particles in the cytoplasms; (c) shows a similar severe cytopathic cell: the squared area is enlarged in (d) with isolated viral particles. (e) BAL cells immunoreacting with anti-spike antibodies were variably represented in BAL samples (10x), including (f) ciliated epithelia, 20x. Scale bar = 2 µm and 200 nm.


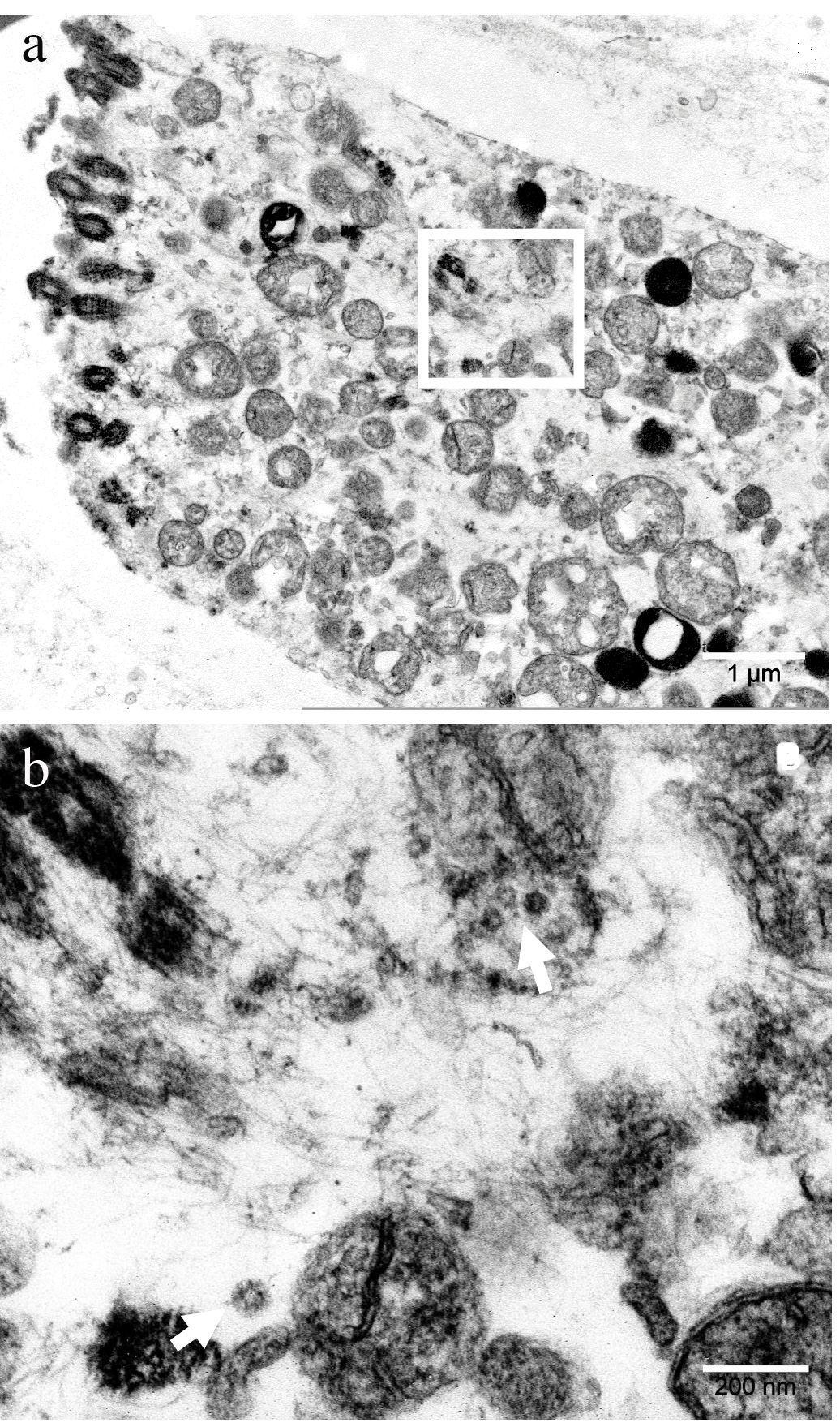


Figure S2. (a-b) Electron micrograph showing a ciliated cytopathic cell with single viral particles (enlarged in b) and vesicles containing smaller viral particles (upper white arrow in b). Scale bar = 1 µm and 200 nm


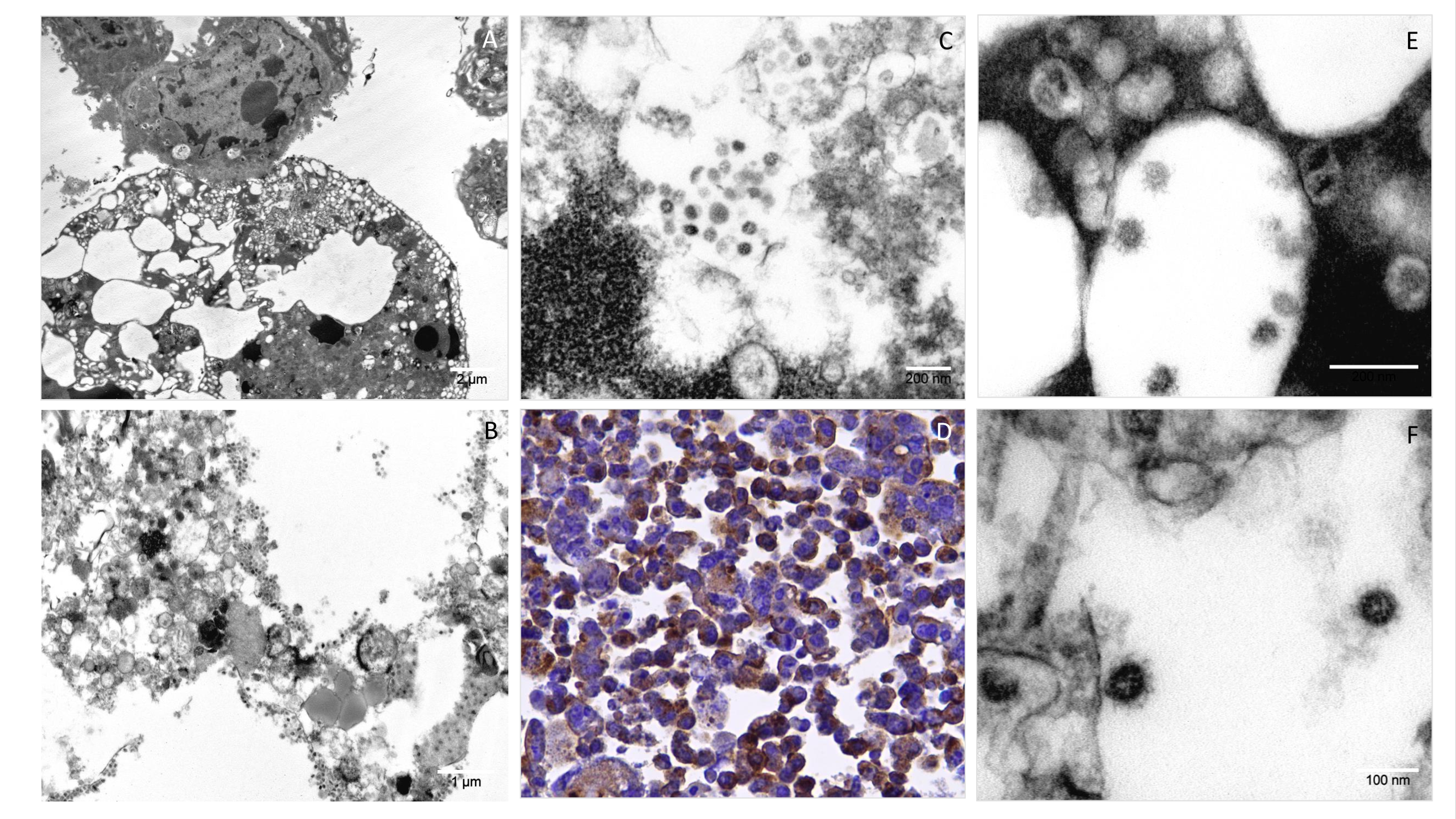


Figure S3. The figure shows infected VERO E6 cells at (a) 48 and (b-f) 72 hours from infection with SARS-CoV-2. (a) severely cytopathic cell (lower) in close contact with a non-cytopathic cell (upper). Scale bar = 2 µm. (b) Extensively damaged cell with multiple viral arrays. Scale bar = 1 µm. (c) Viral particles with variable morphology, with and without spikes in cytoplasmic vesicles (🡪) and free in the cytoplasm (squared area). Scale bar = 200 nm. (d) the extensive immunostaining with anti-spike antibodies in infected VERO E6 cells, 20x. (e) Spiked viral particles in cytoplasmic vesicles. Scale bar = 200 nm. (f) Spiked viral particles free in the cytoplasm. Scale bar = 100 nm.

Table S1. Correlation analysis of entire BAL collected between cell populations and cytokines.

|  | IL6 | | IL8 | | IL10 | |
| --- | --- | --- | --- | --- | --- | --- |
|  | *r* | *p* | *r* | *p* | *r* | *p* |
| MACROPHAGES (%) | -0.4843 | 0.0090 | -0.6555 | 0.0002 | 0.09727 | 0.6224 |
| NEUTROPHILS (%) | 0.3746 | 0.0495 | 0.7921 | < 0.0001 | -0.1518 | 0.4407 |
| LYMPHOCYTES (%) | -0.02908 | 0.8832 | -0.5366 | 0.0032 | 0.2411 | 0.2165 |

r = Spearman coefficient; p = p value

Table S2. Correlation analysis between cytokines quantified in BALs overall.

|  | IL6 | | IL8 | | IL10 | |
| --- | --- | --- | --- | --- | --- | --- |
|  | *r* | *P* | *r* | *p* | *r* | *p* |
| IL6 | - | - | 0.458 | 0.003 | -0.234 | 0.140 |
| IL8 | 0.458 | 0.003 | - |  | -0.448 | 0.003 |
| IL10 | -0.234 | 0.140 | -0.448 | 0.003 |  |  |

r = Spearman coefficient; p = p value


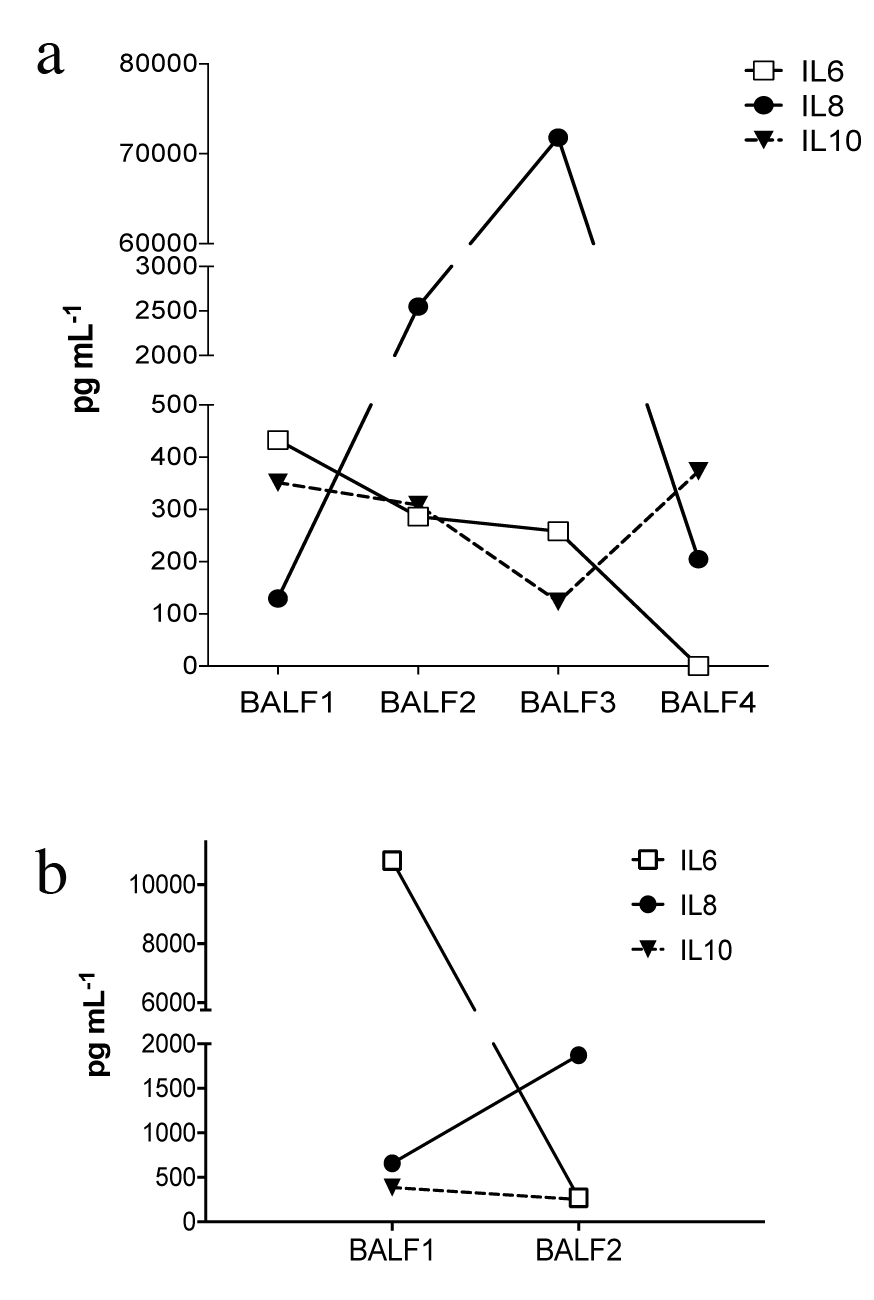


Figure S4. IL6, 8 and 10 trends in two representative ICU patients. (a) survivor (treated with anti-viral + corticosteroids) and (b) non-survivor (treated only with tocilizumab).


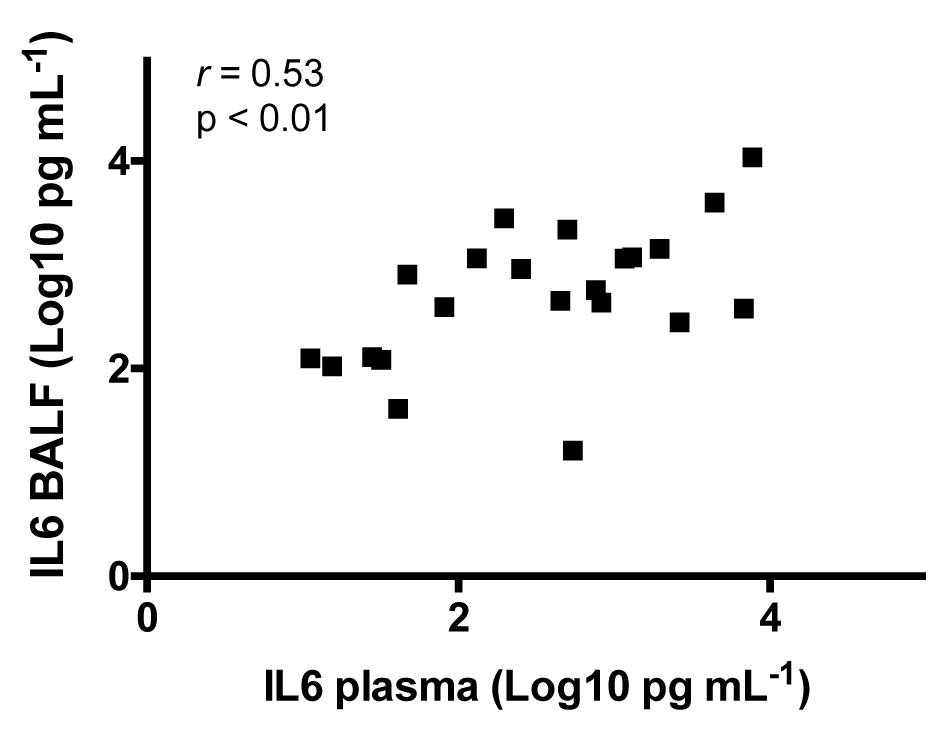


Figure S5. Correlation between IL6 quantified in plasma vs. IL6 quantified in BAL of the same patients sampled at the same day. r = Sparman coefficient; p = p value.
